# Macrophage migration inhibitory factor may play a protective role in osteoarthritis

**DOI:** 10.1186/s13075-021-02442-w

**Published:** 2021-02-20

**Authors:** Ming Liu, Zikun Xie, Guang Sun, Liujun Chen, Dake Qi, Hongwei Zhang, Jieying Xiong, Andrew Furey, Proton Rahman, Guanghua Lei, Guangju Zhai

**Affiliations:** 1 Division of Biomedical Sciences (Genetics), Faculty of Medicine, Memorial University of Newfoundland, St. John’s, NL A1B 3V6 Canada; 2 Department of Orthopaedics, Xiangya Hospital, Central South University, Changsha, China; 3 Discipline of Medicine, Faculty of Medicine, Memorial University of Newfoundland, St. John’s, Canada; 4 College of Pharmacy, University of Manitoba, Winnipeg, Manitoba Canada; 5 Discipline of Surgery, Faculty of Medicine, Memorial University of Newfoundland, St. John’s, Canada

**Keywords:** Macrophage migration inhibitory factor, Osteoarthritis, Inflammation, Cytokines

## Abstract

**Background:**

Osteoarthritis (OA) is the most prevalent form of arthritis and the major cause of disability and overall diminution of quality of life in the elderly population. Currently there is no cure for OA, partly due to the large gaps in our understanding of its underlying molecular and cellular mechanisms. Macrophage migration inhibitory factor (MIF) is a procytokine that mediates pleiotropic inflammatory effects in inflammatory diseases such as rheumatoid arthritis (RA) and ankylosing spondylitis (AS). However, data on the role of MIF in OA is limited with conflicting results. We undertook this study to investigate the role of MIF in OA by examining *MIF* genotype, mRNA expression, and protein levels in the Newfoundland Osteoarthritis Study.

**Methods:**

One hundred nineteen end-stage knee/hip OA patients, 16 RA patients, and 113 healthy controls were included in the study. Two polymorphisms in the *MIF* gene, rs755622, and -794 CATT_5-8_, were genotyped using polymerase chain reaction–restriction fragment length polymorphism (PCR-RFLP) and PCR followed by automated capillary electrophoresis, respectively. *MIF* mRNA levels in articular cartilage and subchondral bone were measured by quantitative polymerase chain reaction. Plasma concentrations of MIF, tumor necrosis factor-alpha (TNF-α), interleukin-6 (IL-6), and interleukin-1 beta (IL-1β) were measured by enzyme-linked immunosorbent assay.

**Results:**

rs755622 and -794 CATT_5-8_ genotypes were not associated with *MIF* mRNA or protein levels or OA (all *p* ≥ 0.19). *MIF* mRNA level in cartilage was lower in OA patients than in controls (*p* = 0.028) and RA patients (*p* = 0.004), while the levels in bone were comparable between OA patients and controls (*p* = 0.165). MIF protein level in plasma was lower in OA patients than in controls (*p* = 3.01 × 10^−10^), while the levels of TNF-α, IL-6 and IL-1β in plasma were all significantly higher in OA patients than in controls (all *p* ≤ 0.0007). Multivariable logistic regression showed lower MIF and higher IL-1β protein levels in plasma were independently associated with OA (OR per SD increase = 0.10 and 8.08; 95% CI = 0.04–0.19 and 4.42–16.82, respectively), but TNF-α and IL-6 became non-significant.

**Conclusions:**

Reduced *MIF* mRNA and protein expression in OA patients suggested MIF might have a protective role in OA and could serve as a biomarker to differentiate OA from other joint disorders.

## Background

Osteoarthritis (OA), the most common form of arthritis, is characterized by the loss of articular cartilage due to an imbalance between extracellular matrix destruction and repair, accompanied with osteophyte formation at joint margins and changes in other joint tissues [1]. OA causes pain and functional limitations in affected joints and is the major cause of disability and overall diminution of quality of life in the elderly population [2]. At present, there is no cure for it yet, partly because of the large gaps in our understanding of the underlying molecular and cellular mechanisms of OA.

Risk factors such as older age, female sex, obesity, and joint injury have been associated with OA [3]. Chondrocyte, the only cellular component in articular cartilage, synthesizes cartilage matrix proteins such as collagens and proteoglycans and maintains the structural and functional integrity of the matrix. Chondrocyte plays a critical role in the pathogenesis of OA in responding to excess or abnormal mechanical loading and biological stimuli such as dysregulated cytokine activities [4]. In OA, excess pro-inflammatory cytokines are produced by joint tissue cells including chondrocytes and released into synovial fluid, and then act on the resident cells in an autocrine-paracrine manner [5]. In response to the elevated level of pro-inflammatory cytokines, the expression of proteinases such as metalloproteinases (MMPs) and aggrecanases is upregulated, and compensatory synthesis pathways are downregulated in chondrocytes, which leads to degradation of cartilage matrix and deterioration in the structural and functional properties of cartilage [4]. The cartilage breakdown products released into the synovial fluid promote synovial inflammation, which produces more pro-inflammatory cytokines and proteases and creates a vicious cycle of more cartilage being degraded and subsequently provoking more inflammation [6].

Macrophage migration inhibitory factor (MIF) is a glucocorticoid (GC)-induced mediator that plays a central role in promoting inflammation by overriding the anti-inflammatory action of GCs [7]. Because of this, numerous studies have been conducted to investigate the role of MIF in diseases, particularly autoimmune disorders such as rheumatoid arthritis (RA) and systemic lupus erythematosus (SLE) where GC therapy is required [8]. Studies have found that MIF is overexpressed in serum, synovial fluid and cultured fibroblast-like synoviocytes (FLS) from RA patients compared with those from controls [9], indicating a significant role of MIF in RA. Similarly, MIF is also implicated in the pathogenesis of SLE [10]. Chronic inflammation has been implicated in OA and cytokines such as tumor necrosis factor alpha (TNF-α), interlukin-6 (IL-6), and interlukin-1β (IL-1β) have been reported to be associated with OA [11–13]. However, data on the role of MIF in OA is limited with conflicting results [14–19]. Therefore, we undertook this study to investigate the potential role of MIF in OA by systematically examining *MIF* genotypes, mRNA and protein expressions in a well-established OA cohort—the Newfoundland Osteoarthritis Study (NFOAS) [20].

## Methods

### Study participants

Study participants were derived from the NFOAS, which was initiated in 2011 to study the genetic, biochemical and epigenetic markers of OA [20]. OA and RA patients were all at the end-stage of the disease and underwent total joint replacement (TJR) surgery in St. John’s, Newfoundland and Labrador (NL), Canada, between 2011 and 2017. Controls were from the same population and had no self-reported arthritis at all joints. Diagnosis of OA and RA was based on American College of Rheumatology (ACR) and 2010 ACR/European League Against Rheumatism (EULAR) clinical diagnostic criteria [21–23] and was confirmed by pathological report after TJR surgery. The study was approved by the Health Research Ethics Authority of Newfoundland and Labrador (HREB # 2011.311) and written consent was obtained from all participants.

### Demographic and joint pain data collection

Date of birth and sex data were collected with a general health questionnaire, and age at the time of TJR surgery was then calculated. Weight and height data were retrieved from Eastern Health Meditech Health Care Information System, and body mass index (BMI) was calculated as weight in kg/squared height in meters. Joint pain of OA and RA patients was assessed using the Western Ontario and McMaster Universities Osteoarthritis Index (WOMAC) Likert version 3.0. WOMAC pain subscale scores 0–20, with 0 representing no pain and 20 representing the most severe pain.

### Plasma separation and blood DNA extraction

Whole blood samples were collected into Vacutainer EDTA tubes following at least 8 h of fasting. Plasma was separated by centrifugation at 2000 RPM for 10 min and stored at − 80 °C until analysis. Blood DNA was extracted by modified salting-out method [24]. Briefly, for 8 ml of whole blood, after plasma was removed, 25 ml of red cell lysis buffer (140 mM NH_4_Cl, 17 mM Tris-HCl, pH 7.65) was added and the sample was then incubated at 37 °C in water bath for 10 min. After centrifugation at 2500 RPM for 5 min and removal of supernatant, another 15 ml of red cell lysis buffer was added, followed by incubation and centrifugation. The pellet was then mixed with 3 ml of nuclei lysis buffer (400 mM NaCl, 2 mM EDTA, 10 mM Tris-HCl, pH 8.0) by vortex. Two hundred microliters of 10% SDS and 500 μl of pronase E solution (3 mg/ml pronase E, 1% SDS, 2 mM EDTA) were added and sample was incubated at 37 °C in water bath till the pellet was digested completely. One milliliter of saturated NaCl (6 M) was added and the sample was vigorously mixed for 15 s and then centrifuged at 2800 RPM for 18 min. The supernatant was carefully transferred into a new tube, and two volumes of absolute ethanol was added. The tube was inverted for a few times and precipitated DNA was fished out with a Pasteur pipette and washed with 70% ethanol. DNA pellet was dried at room temperature, dissolved in 500 μl of TE buffer (1 mM EDTA, 10 mM Tris-HCl, pH 8.0), and stored at 4 °C.

### *MIF* genotyping for single nucleotide polymorphism (SNP) -173 G/C and microsatellite -794 CATT_5-8_

A functional SNP in the promoter region of *MIF*, -173 G/C (rs755622), was genotyped by polymerase chain reaction–restriction fragment length polymorphism (PCR-RFLP) method. The 366-bp polymorphic fragment was amplified with forward primer 5′-ACTAAGAAAGACCCGAGGC-3′ and reverse primer 5′-GGGGCACGTTGGTGTTTAC-3′ in a 20 μl reaction containing 100 ng genomic DNA, 0.1 μM forward and reverse primer each, 0.2 mM dNTPs, 1 mM MgCl_2_, 2 μl 10× PCR buffer, and 0.4 unit of Platinum Taq DNA polymerase (Invitrogen, Waltham, MA, USA, Cat # 10966034). Cycling conditions were as follows: initial activation at 95 °C for 10 min, followed by 95 °C for 1 min, 58 °C for 45 s, 72 °C for 45 s, repeated in 35 cycles, and a final extension at 72 °C for 7 min. Five microliters of PCR product was digested with 5 units of *Alu* I restriction endonuclease (Invitrogen, Waltham, MA, USA, Cat # 45200029) in a 20 μl reaction at 37 °C overnight. The digestion product was resolved on a 2% agarose gel, stained with SYBR^TM^ Safe, and visualized using ultraviolet (UV) gel imaging system. The C allele that contains two *Alu* I cleavage sites yielded 205-, 98-, and 63-bp bands, while the G allele that contains one *Alu* I cleavage site yielded 268- and 98-bp bands.

Microsatellite -794 CATT_5-8_, also located in the promoter region of *MIF*, was genotyped by PCR followed by automated capillary electrophoresis. The forward primer was 5′-CTATGTCATGGCTTATCTTC-3′ labeled with 6-carboxyfluorescein (6-FAM) at 5′-end, and the reverse primer was 5′-TCCACTAATGGTAAACTCGG-3′. PCR reaction and cycling conditions were as above with 200 ng genomic DNA, 1.25 mM MgCl_2_, and annealing temperature 55 °C. PCR product was 1:10 diluted with ddH_2_O and sequenced on ABI 3730 DNA Analyzer (Applied Biosystems, Waltham, MA, USA). The CATT_5-8_ alleles were identified using Peak Scanner software V 1.0 (Applied Biosystems, Waltham, MA, USA).

### RNA extraction and *MIF* gene expression measurement

Articular cartilage and subchondral bone tissues of OA patients and articular cartilage tissue of RA patients were retained from either tibial plateaus or femoral heads during TJR surgeries; those of controls were collected from femoral heads during hemiarthroplasty for hip fracture patients. Samples were flash-frozen and stored in liquid nitrogen until the experiment. RNA was extracted from the cartilage and bone samples as described previously [25]. mRNA levels of the *MIF* gene were measured by quantitative PCR (qPCR) method. Complementary DNA (cDNA) synthesis, qPCR, and relative quantification (RQ) of *MIF* gene expression were performed as described previously [25]. Table 1 presents the primer sequences, the sizes of PCR products, as well as primer efficiencies.

**Table 1:** Primers used for *MIF* qPCR

Gene	Primer sequence (5′ > 3′)	Amplicon Size (bp)	Efficiency	***R***^**2**^
*GAPDH*	Forward: GCAAATTCCATGGCACCGT	106	102%	0.999
	Reverse: TCGCCCCACTTGATTTTGG			
*MIF*	Forward: CCCGGACAGGGTCTACATCAACTA	64	96%	0.996
	Reverse: GGAGTTGTTCCAGCCCACAT			

*qPCR* quantitative polymerase chain reaction, *GAPDH* glyceraldehyde-3-phosphate dehydrogenase, *MIF* macrophage migration inhibitory factor

### Enzyme-linked immunosorbent assay (ELISA) of MIF, TNF-α, IL-6, and IL-1β

Plasma levels of MIF, TNF-α, IL-6, and IL-1β were measured in duplicates using sandwich enzyme-linked immunosorbent assay (ELISA) kits (Human MIF DuoSet ELISA, DY289; DuoSet ELISA Ancillary Reagent Kit 2, DY008; Human TNF-alpha Quantikine HS ELISA, SSTA00E; Human IL-6 Quantikine HS ELISA Kit, SS600C; Human IL-1 beta Quantikine HS ELISA Kit, SSLB00D, R&D systems, Minneapolis, MN, USA). All assays were performed according to the manufacturer’s instructions. The intra-assay CV% of MIF, TNF-α, IL-6, and IL-1β were ≤ 2.5%, 2.2%, 4.7%, and 4.4%, respectively, and inter-assay CV% were ≤ 6.7%, 10.8%, and 10.7% for TNF-α, IL-6, and IL-1β, respectively.

### Statistical analysis

Normality of distribution was tested with the Shapiro-Wilk test. Age, BMI, and WOMAC pain scores, which were not normally distributed, were compared with the Mann-Whitney *U* test. Sex distribution was tested with chi-squared test. Hardy–Weinberg equilibrium was assessed by Fisher’s exact test. Association of *MIF* genotypes with OA was tested by chi-squared test of the distribution of genotypes containing risk alleles, -173 C allele in rs755622 and 7 or above CATT-repeats in both alleles in -794 CATT_5-8_ [26]. *MIF* mRNA levels were natural log transformed and compared with independent sample Student’s *t* test. Plasma cytokine levels were natural log transformed and compared with the Mann-Whitney *U* test due to their non-normal distribution. Linear regression was used to test associations between *MIF* genotypes and mRNA and protein levels. Spearman correlation coefficients were calculated to evaluate the relationships between the levels of MIF and other pro-inflammatory cytokines in plasma. Logistic regression was utilized for adjustment for potential confounding factors. Natural log-transformed cytokine concentrations were standardized using *Z* score for the logistic regression. Significant level was defined at *α* = 0.05. All analyses were performed in R Studio with R version 3.6.3. Visualizations of the results were done with ggplot2 R package [27].

## Results

A total of 248 study participants were included in the study, of which 119 were OA patients, 16 were RA patients, and 113 were controls. OA patients were significantly older than controls, had a higher BMI, and less females than controls and RA patients (all *p* < 0.04, Table 2). OA patients were also older than RA patients, but the difference was not statistically significant (*p* > 0.05, Table 2). There was no difference in age, BMI, or percentage of females between controls and RA patients (all *p* ≥ 0.13, Table 2).

**Table 2:** Characteristics of the study participants*

	Control (***n*** = 113)	OA (***n*** = 119)	RA (***n*** = 16)	***p*** value		
				OA vs. control	RA vs. control	OA vs. RA
**Age (years)**	58.08 ± 6.60	65.51 ± 8.90	59.34 ± 10.21	1.94 × 10^−11^	0.202	0.051
**Sex (% of females)**	61.95%	47.06%	81.25%	0.023	0.131	0.010
**BMI (kg/m**^**2**^**)**	30.02 ± 4.91	32.11 ± 6.26	28.75 ± 6.76	0.036	0.129	0.023

*BMI* body mass index, *OA* osteoarthritis, *RA* rheumatoid arthritis

*Values are the mean ± SD unless indicated otherwise. *P* values were obtained by the Mann-Whitney *U* test or chi-squared test wherever appropriate

### *MIF* genotypes and OA

Two polymorphisms in the promoter region of *MIF* were genotyped for OA patients and controls. For rs755622, the overall frequency of GG genotype was 76%, and that of CC was 2%. The distribution of the genotypes showed no deviation from the Hardy-Weinberg equilibrium (*p* = 0.56). Minor allele frequency (MAF) was 13%, which was lower than reported in other Caucasian populations [28]. Frequency of genotypes containing C allele (GC + CC) was 20% in control group and 28% in OA group, but the difference was not statistically significant (*p* = 0.19). When examining hip (*n* = 64) and knee (*n* = 55) separately, GC + CC frequency was 28% in hip OA and 27% in knee OA patients, and it was not associated with OA in either hip or knee joint (*p* = 0.24 and 0.31 for hip and knee OA, respectively).

Six genotypes of -794 CATT_5-8_ were identified, 6/6, 6/7, 6/8, 7/7, 7/8, and 8/8. The frequencies were 8%, 33%, 3%, 41%, 15%, and 1%, respectively, which were also in accordance with the Hardy–Weinberg equilibrium (*p* = 0.35). Allele frequencies were 26%, 65%, and 10% for CATT_6_, CATT_7_, and CATT_8_. Compared to what was reported in other Caucasian populations [29], CATT_5_ allele was absent in our cohort, CATT_6_ had a lower frequency, while CATT_7_ and CATT_8_ had much higher frequencies. Frequency of genotypes containing 7 or above CATT-repeats in both alleles (7/7+7/8+8/8) was 56% in control group, 57% in OA group, 63% in hip OA, and 51% in knee OA. No significant association between -794 CATT_5-8_ and OA was observed (*p* = 0.83, 0.38 and 0.55 for OA, hip OA and knee OA, respectively). Genotype frequencies of rs755622 and -794 CATT_5-8_ in control and OA groups were presented in Table 3.

**Table 3:** Genotype frequencies of rs755622 and -794 CATT_5-8_ polymorphisms in control and OA groups

Genotype	Control	OA		Hip OA		Knee OA	
	***n*** = 113 (%)	***n*** = 119 (%)	***p*** value*	***n*** = 64 (%)	***p*** value*	***n*** = 55 (%)	***p*** value*
rs755622			0.19		0.24		0.31
GG	90 (79.6)	86 (72.3)		46 (71.9)		40 (72.7)	
GC	20 (17.7)	31 (26.1)		17 (26.6)		14 (25.5)	
CC	3 (2.7)	2 (1.7)		1 (1.6)		1 (1.8)	
-794 CATT_5-8_			0.83		0.38		0.55
6/6	8 (7.1)	10 (8.4)		4 (6.3)		6 (10.9)	
6/7	41 (36.3)	35 (29.4)		16 (25)		19 (34.5)	
6/8	1 (0.9)	6 (5.0)		4 (6.3)		2 (3.6)	
7/7	48 (42.5)	47 (39.5)		30 (46.9)		17 (30.9)	
7/8	13 (11.5)	21 (17.6)		10 (15.6)		11 (20)	
8/8	2 (1.8)	0 (0)		0 (0)		0 (0)	

*MIF* macrophage migration inhibitory factor, *OA* osteoarthritis

*P values for rs755622 were obtained by chi-squared test of the distribution of genotypes containing C allele (GG vs. GC + CC); *p* values for -794 CATT_5-8_ were obtained by chi-squared test of the distribution of genotypes containing 7 or above CATT-repeats in both alleles (6/6+6/7+6/8 vs. 7/7+7/8+8/8)

### *MIF* mRNA levels in joint cartilage/bone and OA

**Fig. 1:**
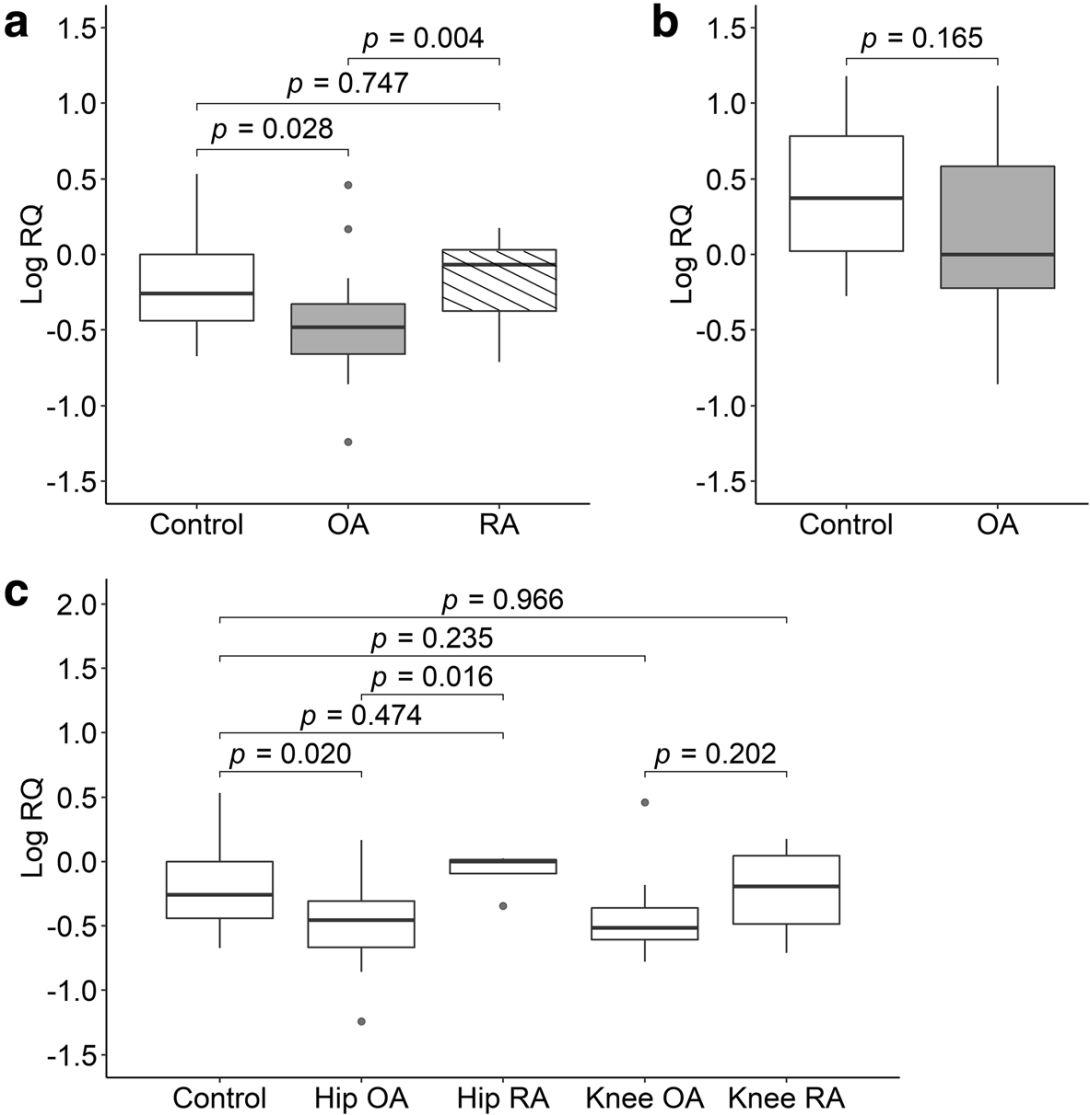
Boxplots for natural log-transformed *MIF* mRNA levels in control, OA, and RA groups. **a** Natural log-transformed *MIF* mRNA levels in control, OA, and RA groups in cartilage. **b** Natural log-transformed *MIF* mRNA levels in control and OA groups in subchondral bone. **c** Natural log-transformed *MIF* mRNA levels in control, hip OA, hip RA, knee OA, and knee RA groups in cartilage. MIF, macrophage migration inhibitory factor; OA, osteoarthritis; RA, rheumatoid arthritis; RQ, relative quantification; *p* values were obtained from independent sample Student’s *t* test

*MIF* mRNA levels were assessed in a total of 59 articular cartilage and 43 subchondral bone samples including 13 control, 30 OA and 16 RA articular cartilage samples, and 6 control and 37 OA subchondral bone samples. The RA samples were included so that we could compare the difference between OA and an autoimmune disease that also involves chronic inflammation. In cartilage, natural log-transformed *MIF* mRNA level was significantly lower in OA patients than in controls (mean ± SD, logRQ = − 0.47 ± 0.32 vs. − 0.21 ± 0.35; *p* = 0.028) and RA patients (mean ± SD, logRQ = − 0.18 ± 0.28; *p* = 0.004). The levels were not significantly different between RA patients and controls (*p* = 0.747). When hip and knee joints were examined separately, the significances remained between hip OA patients (*n* = 21) and controls (mean ± SD, logRQ = − 0.49 ± 0.30 vs. - 0.21 ± 0.35; *p* = 0.02) and hip RA patients (*n* = 4; mean ± SD, logRQ = − 0.08 ± 0.18; *p* = 0.016). Knee OA patients (*n* = 9) also had a lower level of *MIF* mRNA than controls and knee RA patients (*n* = 12) (mean ± SD, logRQ = − 0.40 ± 0.38, − 0.21 ± 0.35, and − 0.21 ± 0.30, respectively), but the differences were not statistically significant (*p* ≥ 0.2). Natural log-transformed *MIF* mRNA levels were not significantly different between OA patients and controls in subchondral bone samples (mean ± SD, logRQ = 0.11 ± 0.47 vs. 0.41 ± 0.56; *p* = 0.165), which were all from hip joints (Fig. 1). There was no correlation between natural log-transformed *MIF* mRNA level and the two polymorphisms in *MIF* promoter region (*p* = 0.97 for rs755622 and *p* = 0.93 for -794 CATT_5-8_).

### Association between plasma MIF levels and OA

**Fig. 2:**
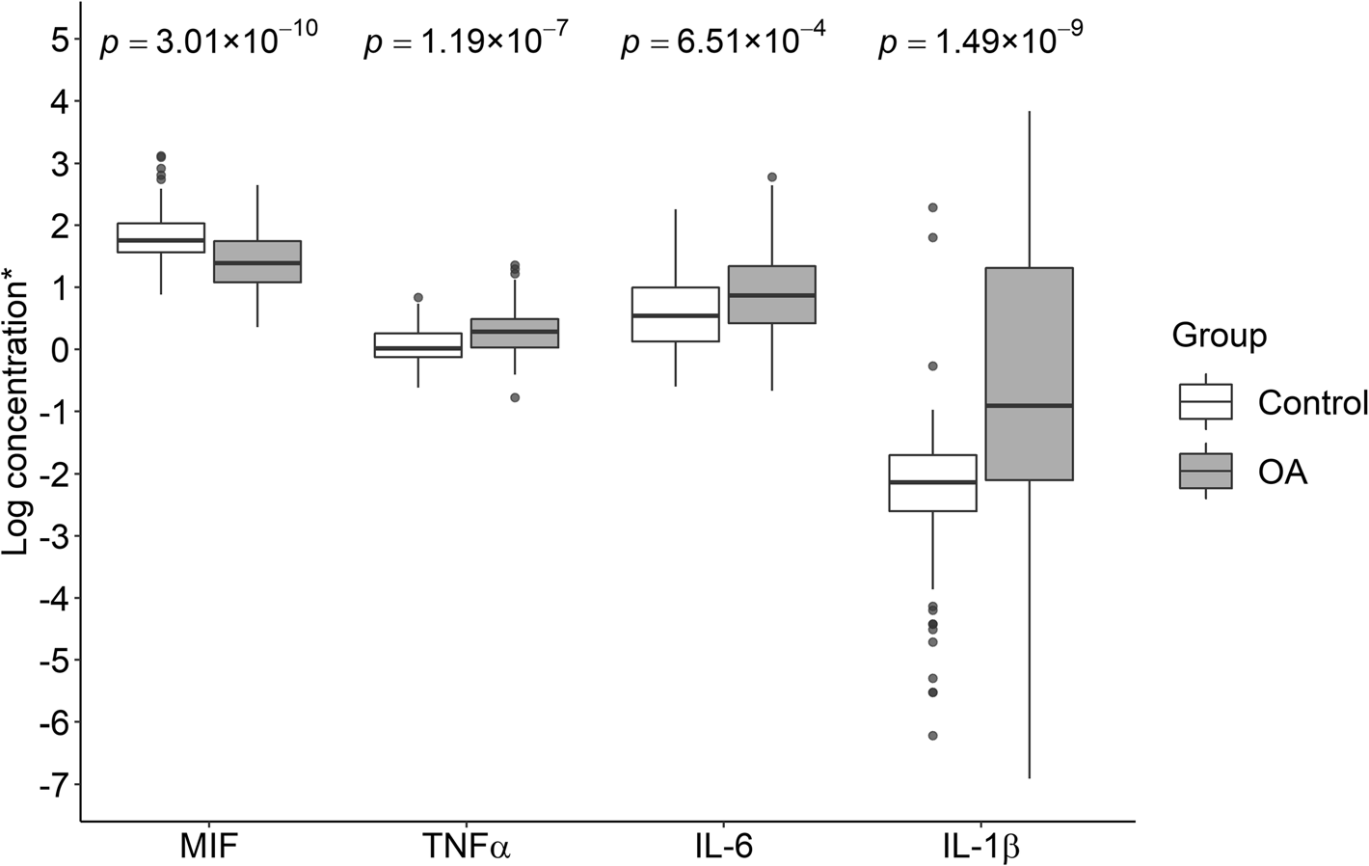
Boxplots for natural log-transformed plasma cytokine levels. MIF, macrophage migration inhibitory factor; TNF-a, tumor necrosis factor alpha; IL-6, interlukin-6; IL-1β, interlukin-1β; OA, osteoarthritis. Log concentration for MIF: log ng/ml; log concentration for TNF-a, IL-6, and IL-1β: log pg/ml; *p* values were obtained from Mann-Whitney *U* test

Plasma MIF concentrations were measured for all 119 OA patients and 109 controls. The raw MIF concentration was natural log-transformed for normality because its distribution was highly skewed. The average natural log-transformed MIF level in OA patients was significantly lower than that in controls (mean ± SD, 1.43 ± 0.46 vs. 1.82 ± 0.40 log ng/ml; *p* = 3.01 × 10^−10^, Fig. 2). The significances remained when hip and knee OA were compared with control group separately (mean ± SD, 1.46 ± 0.46 and 1.40 ± 0.45 log ng/ml in hip and knee OA, respectively; *p* = 1.05 × 10^−6^ and 3.37 × 10^−8^, respectively). Natural log-transformed plasma MIF level was not correlated with *MIF* genotypes (*p* = 0.31 for rs755622 and *p* = 0.62 for -794 CATT_5-8_) or natural log-transformed *MIF* mRNA level in cartilage (*p* = 0.21).

### Relationships between joint pain intensity and MIF levels and OA

Among the 30 OA and 16 RA patients with cartilage *MIF* mRNA level data, 29 OA and 11 RA patients had WOMAC pain scores available, ranging from 2 to 20. There was no correlation between cartilage *MIF* mRNA levels and WOMAC pain scores (*p* = 0.72). Among the 119 OA patients with plasma MIF concentration data, 111 had WOMAC pain scores available, also ranging from 2 to 20. There was no correlation between plasma MIF concentrations and WOMAC pain scores (*p* = 0.26). WOMAC pain scores were not significantly different between OA and RA patients (13.98 ± 4.55 vs. 15.36 ± 2.94, *p* = 0.44).

### Relationships between OA, TNF-α, IL-6, IL-1β, and MIF

Given that MIF promotes the production of cytokines [7], we measured the levels of three inflammatory factors, namely TNF-α, IL-6, IL-1β in the same plasma samples. We found that the natural log-transformed concentrations of the three cytokines in OA patients were all significantly higher than those in controls (all *p* ≤ 0.0007) (Fig. 2). MIF was significantly correlated with IL-1β (rho = 0.18, *p* = 0.01) but not TNF-α or IL-6 (*p* > 0.2), and TNF-α, IL-6, and IL-1β were correlated with each other (*p* ≤ 0.01).

In the multivariable analysis with logistic regression including all four cytokines and age, sex, and BMI, we found that lower MIF and higher IL-1β natural log-transformed concentrations in plasma were independently associated with OA (OR per SD increase = 0.10 (95% CI = 0.04–0.19) and 8.08 (95% CI 4.42–16.82), respectively), while TNF-α and IL-6 became non-significant.

## Discussion

We investigated the role of MIF in OA by systematically examining the association between OA and *MIF* genotypes, mRNA expression, and protein production and found that MIF might have a protective role in OA.

rs755622 and -794 CATT_5-8_ are two polymorphisms located in the promoter region of the *MIF* gene, and studies on their association with *MIF* expression have yielded conflicting results. *Radstake TR* et al. reported that rs755622 C and -794 CATT_7_ alleles were associated with higher MIF level in circulation in the Netherlands population [26], while *Baños-Hernández CJ* et al. and *Cruz-Mosso* et al. found that there was no association between these alleles and blood *MIF* mRNA level or serum MIF level in the southern Mexican population [30, 31]. In consistent with *Baños-Hernández* and *Cruz-Mosso*’s findings, we did not observe any association between these variants and *MIF* expression either at mRNA level in cartilage or protein level in plasma, suggesting the function of these two variants may be population specific. The *MIF* allelic structure was reported to have significant population stratification [32]. *Zhong X* et al. compared allele frequencies of -794 CATT_5-8_ in five different populations including Zambia, North African, Korean, Caucasian, and African American and found they were highly variable between populations, likely due to relatedness between populations and selection pressure [32]. Similarly, the allele frequencies of rs755622 between Caucasian, African, and African American also showed significant variation [29, 32]. Our study cohort had a lower MAF of rs755622, absence of CATT_5_ allele, lower frequency of CATT_6_ allele and higher frequencies of CATT_7-8_ alleles for -794 CATT_5-8_ compared to what was reported in other Caucasian populations [28, 29], supporting this and suggesting different regulatory mechanisms for *MIF* expression in the NL population which has an unique genetic structure as a genetic isolate [33].

These two polymorphisms have been associated with the susceptibility or severity of different diseases. rs755622 C and -794 CATT_7_ alleles were found to be associated with increased risk of RA [34], systemic sclerosis (SSc) [30], and SLE [31], and RA patients with rs755622 C allele and homozygotes for -794 CATT_7_ allele displayed higher levels of joint damage [26]. The current study was the first study to examine these variants in OA, and in contrast to what was reported in autoimmune diseases such as RA and SLE, we found neither of these variants was associated with OA.

However, while *MIF* mRNA level in cartilage was not correlated with MIF protein level in plasma, we found that *MIF* mRNA level in articular cartilage was significantly lower in OA patients than in controls and RA patients, and MIF protein level in plasma was also significantly lower in OA patients than in controls. While there was no data reported on *MIF* mRNA expression in human cartilage, several studies reported MIF protein levels in synovial fluid samples. Most of these studies did not have healthy controls but compared between OA and other autoimmune disease such as ankylosing spondylitis (AS) [14] and RA [15]. Consistent with our findings, they reported a significant lower level of MIF protein in OA patients than in AS and RA patients [14, 15], suggesting a different role of MIF between OA and autoimmune diseases. However, we found that there was no difference in *MIF* mRNA level in cartilage between RA patients and controls. This is in contrast to what was found in synovial fluid [35], suggesting that cartilage may not be a major contributor of MIF production in RA; rather, MIF may be released by other joint tissues such as synovium into synovial fluid and cause cartilage degradation. Further studies are needed to confirm this.

Previous reports on blood MIF level in OA were conflicting. While *Liu M & Hu C* found that serum level of MIF was significantly higher in radiographic OA patients than in controls and was associated with radiographic severity of the disease [18], *Zhang PL* et al. reported the level was similar between OA and healthy controls [17]. The discrepancy between these reports and our findings is unclear but could be partially explained by the difference in study populations. In addition to the difference in disease stage, these previous studies were from East Asia whereas our study population was Caucasian. Further, *Zhang PL* et al. [17] showed MIF levels in synovial fluid but not in serum were associated with the severity of self-reported pain in OA patients. We found no association between the WOMAC pain severity score and MIF protein level in plasma, in agreement with their findings that compared to that in the circulating system, the alteration of MIF concentration on a local level might have a closer relationship with the pain severity of the joints. We also found no association between the WOMAC pain severity score and *MIF* mRNA level in cartilage, suggesting a similar scenario to that in RA, that cartilage may not be the main source of MIF in synovial fluid in OA. A *MIF* knock-out mice study [16] showed that the deletion of *MIF* reduced OA severity in aged mice but not in surgical induced OA model, suggesting the role of MIF may be different in different subtypes of OA.

To the best of our knowledge, the current study was the first to have systematically examined the role of MIF in OA and documented evidence that MIF might have a protective role in OA. MIF is an intriguing cytokine which has multiple, sometimes opposite functions depending on the cellular source, the time of release, and the type of disease [14, 36]. When binding to CXCR2 or CXCR4, MIF promotes inflammation [37]; when binding to CD74, it appears to have beneficial effects [38]. MIF can also fine-tune inflammatory microenvironment to maintain homeostasis or a stable state of inflammation [39, 40] and exert organ- and tissue-protective effects during diseases [41, 42]. The beneficial effects of MIF have been reported in diseases such as community-acquired pneumonia [43], experimental liver fibrosis [44], and ischemic heart [38]. While how MIF exerts a beneficial effect in OA remains to be examined, in 3D culture, with MIF added to the medium, the expression of type II collagen (COL2) and aggrecan in chondrocytes was significantly enhanced while type X collagen (COL10) level was unchanged [45]. In mice transplanted with tissue-engineered cartilage from *Mif* +/+ and *Mif* −/− chondrocytes, *Mif* +/+ construct also had higher COL2 expression level but unchanged COL10 level [45]. These results suggested MIF secreted from chondrocytes could promote extracellular matrix (ECM) formation in an autocrine manner without inducing chondrocyte hypertrophy.

In OA joints, cartilage damage results in the production of damage-associated molecular patterns (DAMPs). DAMPs signal through pattern recognition receptors (PRRs) such as toll-like receptors (TLRs) on chondrocytes, synovial macrophages, and FLS to induce production of inflammatory mediators through activation of innate immune system [46]. Cytokines including TNF-α, IL-1β, and IL-6 have been reported to be increased in OA patients [11, 47]. Consistent with these previous reports, we found that the concentrations of all three cytokines in plasma were significantly increased in OA patients compared to controls in the current study. The increased expression of these cytokines can augment production of chondrocyte-mediated catabolic proteinases and inhibit anabolic processes, and thus promote further ECM breakdown, perpetuating the cycle of inflammation and cartilage degradation [4]. MIF is thought to be an upstream regulator in innate immunity, mediates macrophage activation and retention, upregulates the expression of TLR4, and directly or indirectly promotes the production or expression of a large panel of pro-inflammatory molecules including cytokines such as TNF-α, IL-1β, and IL-6 [7, 48]. However, our data showed that MIF was not correlated with TNF-α or IL-6 and only had a moderate correlation with IL-1β. Further, the multivariable analysis showed that both MIF and IL-1β were independently and significantly associated with OA but not TNF-α or IL-6, suggesting that the increased levels of TNF-α, IL-1β, and IL-6 in OA were regulated by different mechanisms rather than MIF.

There are some limitations in the study. Hip and knee OA may have different etiologies. Our results showed that there was no difference in MIF protein levels in plasma between knee and hip OA patients, suggesting that MIF plays a similar role in knee and hip OA. However, when looking at *MIF* mRNA expression in cartilage in knee and hip OA separately, the significance was seen only in hip OA. There was a trend association for knee OA but statistically it was not significant, most likely due to small sample size. Secondly, due to the nature of the cross-sectional analyses, we were not able to assess the causal effect of MIF in OA. Longitudinal or Mendelian randomization studies are required to confirm this. Lastly, all study participants were from genetically homogenous NL population. Given that the allelic structure of *MIF* gene polymorphisms is strongly affected by the population studied, the generalizability of our finding to other populations may be limited.

## Conclusions

Despite the *MIF* mRNA expression in cartilage was not correlated with the plasma MIF protein levels, we observed that both of them were reduced in OA patients, suggesting a protective role of MIF in OA which was different from what was reported in autoimmune disorders such as RA, AS, and SLE. Plasma MIF level could serve as a potential biomarker to differentiate OA from other joint diseases such as RA and AS.

## Data Availability

The datasets used and/or analyzed during the current study are available from the corresponding author on reasonable request.

## Abbreviations

6-FAM: 6-Carboxyfluorescein
ACR: American College of Rheumatology
AS: Ankylosing spondylitis
BMI: Body mass index
cDNA: Complementary DNA
COL10: Type X collagen
COL2: Type II collagen
DAMP: Damage-associated molecular pattern
ECM: Extracellular matrix
ELISA: Enzyme-linked immunosorbent assay
EULAR: European League Against Rheumatism
FLS: Fibroblast-like synoviocytes
GC: Glucocorticoid
IL-1β: Interleukin-1 beta
IL-6: Interleukin-6
MAF: Minor allele frequency
MIF: Macrophage migration inhibitory factor
MMP: Metalloproteinase
NFOAS: Newfoundland Osteoarthritis Study
OA: Osteoarthritis
PCR-RFLP: Polymerase chain reaction–restriction fragment length polymorphism
PRR: Pattern recognition receptor
qPCR: Quantitative PCR
RA: Rheumatoid arthritis
RQ: Relative quantification
SD: Standard deviation
SLE: Systemic lupus erythematosus
SNP: Single nucleotide polymorphism
SSc: Systemic sclerosis
TJR: Total joint replacement
TLR: Toll-like receptor
TNF-α: Tumor necrosis factor-alpha
UV: Ultraviolet
WOMAC: Western Ontario and McMaster Universities Osteoarthritis Index
